# Vitamin K: a potential missing link in critical illness–a scoping review

**DOI:** 10.1186/s13054-024-05001-2

**Published:** 2024-07-01

**Authors:** Michelle Carmen Paulus, Marjolein Drent, Imre Willemijn Kehinde Kouw, Michiel Gerard Juliaan Balvers, Aalt Bast, Arthur Raymond Hubert van Zanten

**Affiliations:** 1 Department of Intensive Care Medicine & Research, Gelderse Vallei Hospital, Willy Brandtlaan 10, 6716 RP Ede, The Netherlands; 2 Division of Human Nutrition and Health, Nutritional Biology, Wageningen University & Research, HELIX (Building 124), Stippeneng 4, 6708 WE Wageningen, The Netherlands; 3 Department of Pharmacology and Toxicology, Faculty of Health, Medicine, and Life Science, Maastricht University, Universiteitssingel 40, 6229 ER Maastricht, The Netherlands; 4 Interstitial Lung Diseases (ILD) Center of Excellence, St. Antonius Hospital, Nieuwegein, Koekoekslaan 1, 3435 CM Nieuwegein, The Netherlands; 5 ILD Care Foundation Research Team, Heideoordlaan 8, 6711NR Ede, The Netherlands

**Keywords:** Vitamin K, Gla protein, PIVKA-II, ICU, Micronutrients

## Abstract

**Background:**

Vitamin K is essential for numerous physiological processes, including coagulation, bone metabolism, tissue calcification, and antioxidant activity. Deficiency, prevalent in critically ill ICU patients, impacts coagulation and increases the risk of bleeding and other complications. This review aims to elucidate the metabolism of vitamin K in the context of critical illness and identify a potential therapeutic approach.

**Methods:**

In December 2023, a scoping review was conducted using the PRISMA Extension for Scoping Reviews. Literature was searched in PubMed, Embase, and Cochrane databases without restrictions. Inclusion criteria were studies on adult ICU patients discussing vitamin K deficiency and/or supplementation.

**Results:**

A total of 1712 articles were screened, and 13 met the inclusion criteria. Vitamin K deficiency in ICU patients is linked to malnutrition, impaired absorption, antibiotic use, increased turnover, and genetic factors. Observational studies show higher PIVKA-II levels in ICU patients, indicating reduced vitamin K status. Risk factors include inadequate intake, disrupted absorption, and increased physiological demands. Supplementation studies suggest vitamin K can improve status but not normalize it completely. Vitamin K deficiency may correlate with prolonged ICU stays, mechanical ventilation, and increased mortality. Factors such as genetic polymorphisms and disrupted microbiomes also contribute to deficiency, underscoring the need for individualized nutritional strategies and further research on optimal supplementation dosages and administration routes.

**Conclusions:**

Addressing vitamin K deficiency in ICU patients is crucial for mitigating risks associated with critical illness, yet optimal management strategies require further investigation.

**Impact research:**

To the best of our knowledge, this review is the first to address the prevalence and progression of vitamin K deficiency in critically ill patients. It guides clinicians in diagnosing and managing vitamin K deficiency in intensive care and suggests practical strategies for supplementing vitamin K in critically ill patients. This review provides a comprehensive overview of the existing literature, and serves as a valuable resource for clinicians, researchers, and policymakers in critical care medicine.

**Supplementary Information:**

The online version contains supplementary material available at 10.1186/s13054-024-05001-2.

## Background

**Fig. 1:**
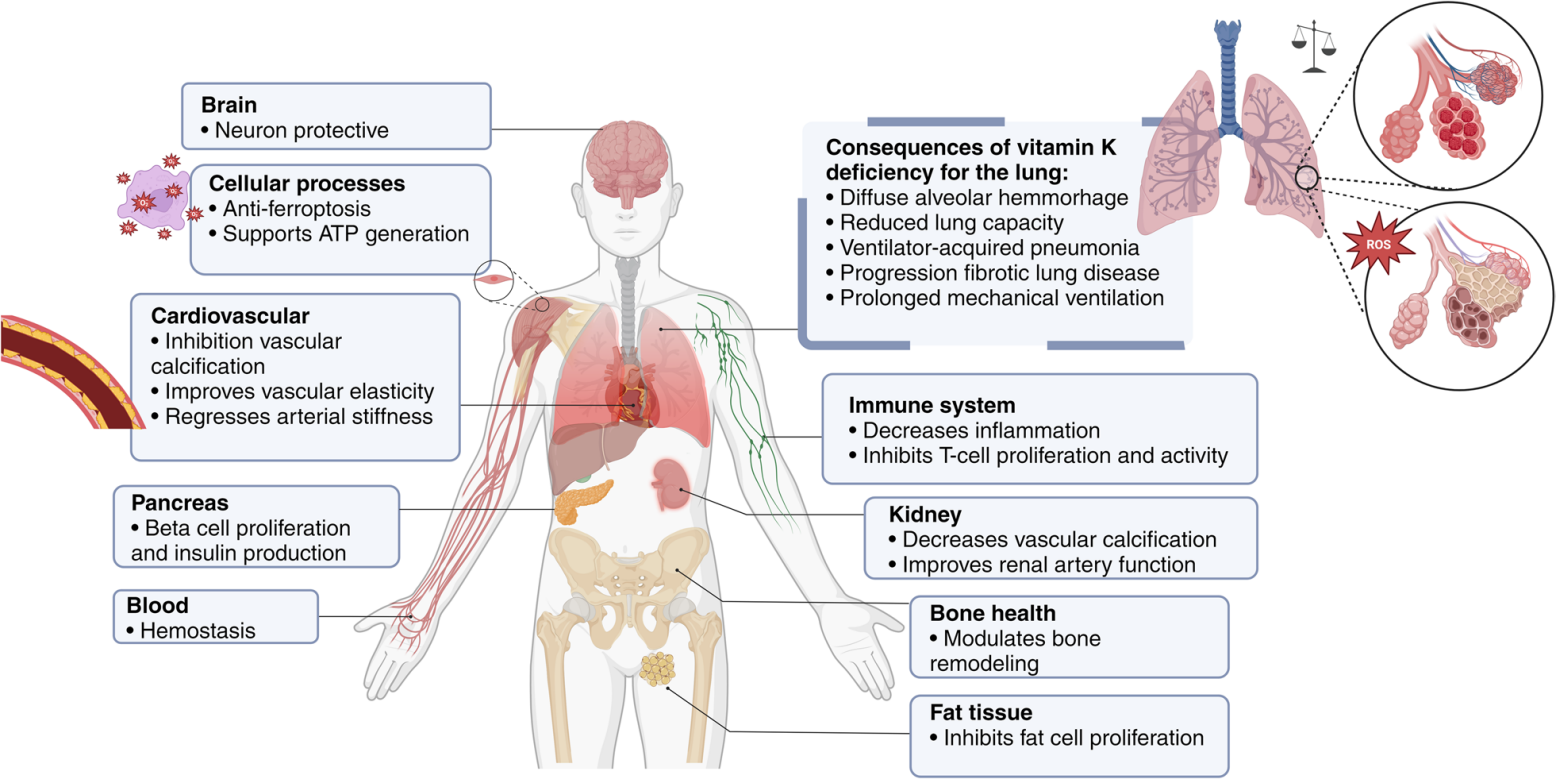
Physiological functions of vitamin K in the body. The diagram demonstrates the numerous roles vitamin K plays in the human body. From facilitating blood clotting in the liver to promoting bone health and cardiovascular function, vitamin K is essential to maintaining overall health. In critical care, vitamin K deficiency can significantly impact patients. Low vitamin K levels impair blood clotting and increase the risk of uncontrolled bleeding, especially when clot formation is critical to the patient's recovery. There is also an increased risk of microbleeding in the lungs, which can lead to diffuse alveolar haemorrhage. This process may also contribute to the development of lung fibrosis by inducing oxidative stress and inflammation. Created with BioRender.com

Vitamin K is critical in various physiological processes, such as hemostasis, low-grade inflammatory diseases, bone metabolism, tissue calcification, and antioxidant activity (Fig. 1). It acts as a cofactor for the enzyme gamma-glutamyl carboxylase (GGCX), which is responsible for the post-translational y-carboxylation of specific glutamic acid (Gla) residues in vitamin K-dependent proteins [1, 2]. Seventeen Gla-proteins with different affinity for GCCX have been identified, exhibiting both hepatic and extrahepatic functions [3, 4].

**Fig. 2:**
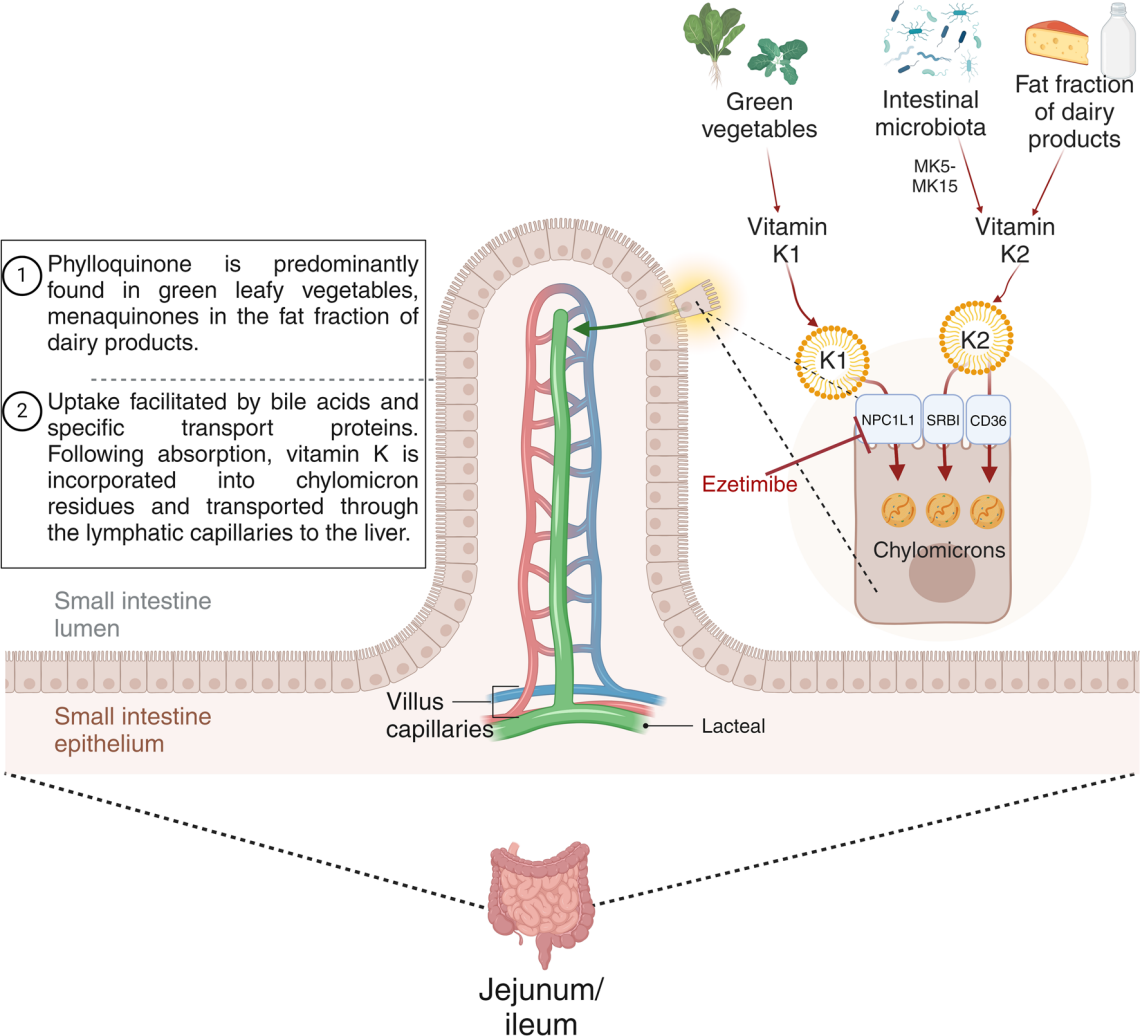
Absorption of vitamin K. Phylloquinone (vitamin K1) is mainly found in green leafy vegetables, while bacteria synthesize menaquinone (vitamin K2), mainly from the fat fraction of dairy products. Vitamin K2 exists in several forms, called MK-n, depending on the side chain. MK-4 can be formed by the conversion of phylloquinone in the intestinal mucosa during absorption or by tissue-specific conversion in the body. Medium- and long-chain MK-n (MK-6 or higher) are synthesized by bacteria and anaerobes in the human colonic microbiota [8]. Vitamin K1 is absorbed in the upper small intestine, particularly in the jejunum and ileum. Vitamin K absorption is facilitated by bile acids and specific transport proteins such as Niemann-Pick C1-like 1 (NPC1L1) and scavenger receptor class B-type I [3]. Following absorption in the small intestine, vitamin K is incorporated into chylomicron remnants and transported through the lymphatic capillaries to the liver [9]. Created with BioRender.com

Vitamin K exists in two naturally biologically active forms. Plants synthesize vitamin K1, also known as phylloquinone, while vitamin K2 encompasses a variety of forms collectively referred to as menaquinones (MK). Vitamin K1 is well-known for its hepatic involvement in coagulation factors II, VII, IX, and X production and anticoagulant proteins C, S, and Z [5]. Protein S is also synthesized extrahepatically in endothelial cells [6]. Vitamin K2 is involved in various processes, including the carboxylation of osteocalcin, matrix Gla protein (MGP), and growth arrest sequence-6 protein (Gas6). Both vitamin K1 and K2 contribute equally to the overall vitamin K status in the body; despite higher vitamin K1 intake, it is less efficiently absorbed [7] (Fig. 2).

**Fig. 3:**
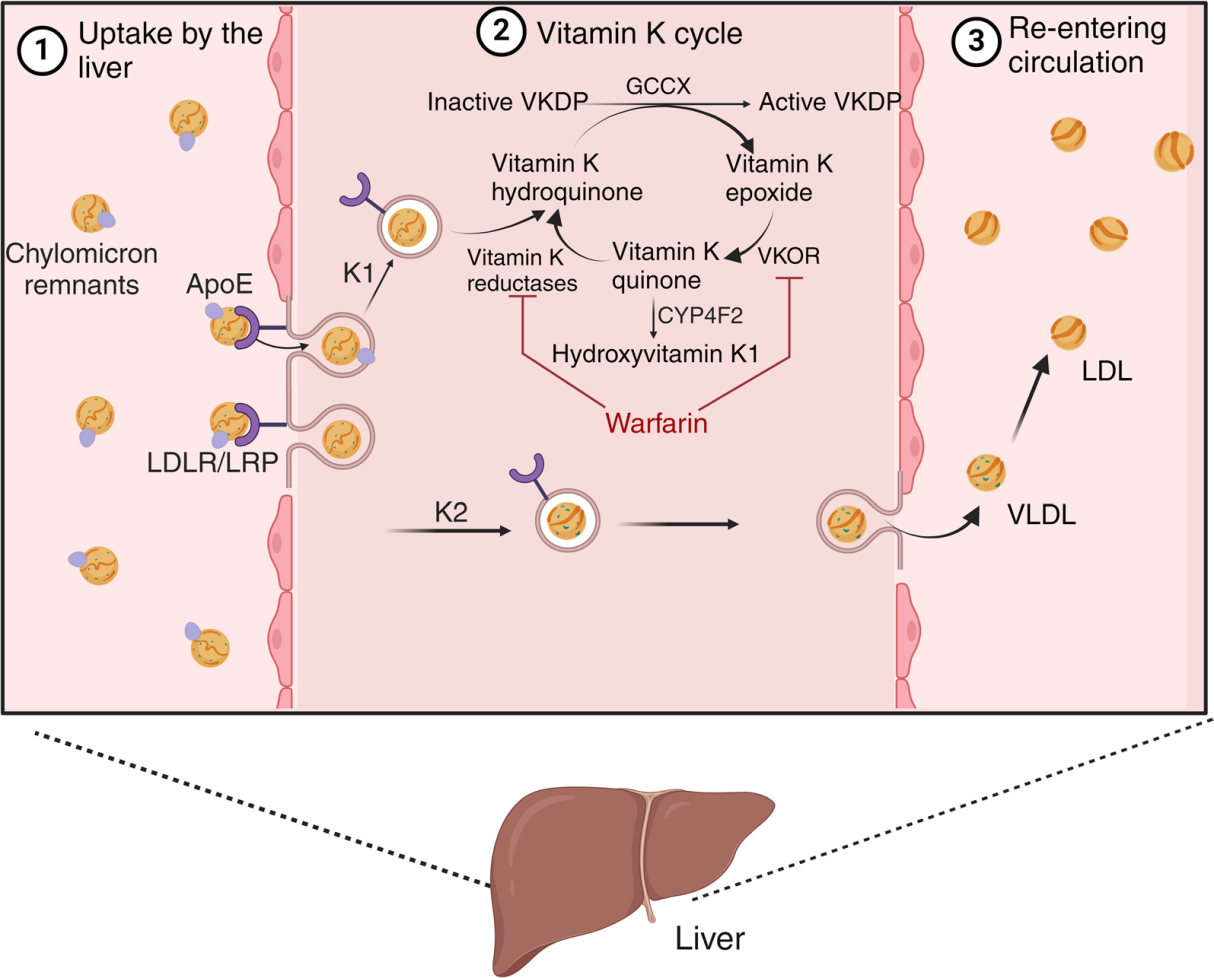
Vitamin K metabolism in the liver. In the liver, vitamin K uptake is regulated by receptor-mediated endocytosis via lipoprotein receptors. Some of it is utilized to synthesize clotting factors, while the remaining amount re-enters the systemic circulation through very low-density lipids. These lipids undergo conversion into low-density lipoproteins (LDL), which serve as carriers for transporting vitamin K to extrahepatic tissues [9]. Initially, vitamin K epoxide (VKO) is converted to vitamin K quinone through vitamin K epoxide reductase (VKOR). Subsequently, vitamin K reductase (VKR) and vitamin K quinone reductases 1 and 2 (VKQR, DT diaphorase) further convert it into VKH. Vitamin K antagonists exert their effect by inhibiting the enzymatic activity of VKOR and VKR, thereby impeding the conversion of vitamin K to its active form. CYP4F2 has been found to play a minor role in the metabolism of vitamin K in its inactive form [11]. This inhibition has implications for both the hepatic and extrahepatic actions of vitamin K [12]. Different cytochrome P450 enzymes are involved in metabolizing coumarins into inactive metabolites. Vitamin K is excreted in the feces via bile and urine. In the absence of warfarin, bile excretion is the predominant route. However, a higher proportion of vitamin K is excreted in the urine when warfarin is used [9]. The different forms of vitamin K have different half-lives. Vitamin K1 and MK-4 have short half-lives of hours, whereas long-chain MK has a much longer half-life of several days [13]. Created with BioRender.com

During the carboxylation of vitamin K-dependent proteins, vitamin K hydroquinone is converted into vitamin K epoxide [10] (Fig. 3). While the body’s vitamin K supply is limited, its effective utilization is facilitated by its capacity for recycling through a two-step reaction, which can occur up to several thousand times [5]. As a result of vitamin K deficiency, the carboxylation process of Gla proteins is impaired, leading to undercarboxylated or uncarboxylated Gla proteins with reduced biological activity. Collectively, these proteins are referred to as "proteins induced in vitamin K absence or antagonism" (PIVKA) (Fig. 3).

Vitamin K deficiency was identified as highly prevalent among critically ill patients, impacting at least 20% of intensive care unit (ICU) patients [14–16]. This scoping review aims to comprehensively explore factors influencing vitamin K deficiency in ICU patients, the impact, potential implications for supplementation strategies, and identifying therapeutic targets.

## Methods

This scoping review was designed using the PRISMA Extension for Scoping Reviews instrument [17], and the protocol was registered in the Open Science Framework (10.17605/OSF.IO/G4Q89). A literature search was conducted to identify all relevant articles exploring vitamin K in critically ill patients on December 20th, 2023, in PubMed, Embase, and Cochrane databases. Essential elements were ‘critically ill’ and 'vitamin K’ (Supplementary Table 1). No restrictions were applied to the year, publication status, or language.

Articles were included if they involved adult ICU patients (age ≥ 18 years) and discussed vitamin K deficiency and/or vitamin K supplementation. No limits were applied to the type of study, the critical care facility type, geographical location, patients’ sex, or race. Relevant systematic reviews and reference lists of included studies were searched to find additional relevant articles, and duplicates were manually checked and removed. Articles were screened via titles and abstracts, and then selected ones had their full text retrieved and reviewed. Disagreements were resolved through discussion until an agreement was reached, and if consensus was not achieved, an adjudicator (AvZ) made the final decision. Supplementary Fig. 1 presents the exclusion criteria for full-text articles. A critical appraisal checklist of the included cross-sectional studies is presented in Supplementary Table 2.

## Results

We found 1,712 potentially relevant studies, of which 121 met the inclusion criteria after screening (Supplementary Fig. 1).

After a full-text review, 13 articles were included, of which six discussed vitamin K deficiency (Table 1) and seven vitamin K supplementation (Table 2). All studies focusing on vitamin K deficiency were observational. The vitamin K supplementation studies consisted of two prospective and four retrospective studies. The independent reviewers unanimously agreed on the included studies.

**Table 1:** Studies assessing Vitamin K deficiency in critically ill patients

Authors and year of publication	Country of origin	Population and sample size	Methodology	Outcome measures/definitions	Key findings
Chakraverty et al. [16]	United Kingdom	N = 235 (87F, 148 M)	Prospective, observational study	Prothrombin time (PT) and Echis time (ET)	134/205 patients (66%) prothrombin time > 1.5
					Retrospective analysis of patients with prolonged PT time (> 1.5): vitamin K deficiency in 9/45 patients (20%)
				Vitamin K deficiency: PT ratio > 1.5 and PT:ET ratio > 1.3 in the absence of alternative cause of coagulopathy	
		Consecutive ICU patients			
					Recommend ICU patients with PT ratio > 2 parenteral vitamin K
Crowther et al. [14]	Canada	N = 40 (20F, 20 M)	Prospective cohort study	Vitamin K deficiency: functional to Echis II ratio < 0,7	7/40 (17.5%) patients had vitamin K deficiency at admission, and 3 patients developed vitamin K deficiency during ICU stay. Functional to Echis time: 7 patients < 0.7 on admission to ICU, 3 patients < 0,7 during ICU stay
		ICU patients with > 3 days ICU stay (not taking vitamin K/vitamin K antagonists)			
				Other outcome measures: INR (> 1.4), D-dimer (> 0.50 μg/ml)	
					Vitamin K administration upon ICU admission might reduce the risk of developing vitamin K deficiency
O’Shaughnessy et al. [114]	United Kingdom	N = 35 (18F, 17 M)	Prospective cohort study	INR (> 1.4), INR/Echis time (normal range 0.8–1.2), PIVKA-II, plasma vitamin K1	Abnormal INR/ET ratio in 16/17 patients with coagulopathy and 0/18 without coagulopathy
				Coagulopathy < 48 h (INR > 1.4)	High PIVKA-II levels were found in 7/17 patients with coagulopathy and 5/18 patients without coagulopathy
				Vitamin K deficiency: vitamin K1 levels and INR/ET ratio (> 1.3) in patients with INR > 1.5	
					PIVKA-II levels and vitamin K levels have discordant results
		Consecutive ICU patients with expected ICU stay > 48 h			Suggest supplementation in patients with malnourishment, critical illness, and receiving broad-spectrum antibiotics
Dahlberg et al. [15]	Sweden	N = 95 (35F, 60 M)	Prospective study	PIVKA-II and PT-INR	Increased PIVKA-II levels at admission and ICU stay (especially in cardiac arrest patients)
		General ICU or postoperative unit			No correlation between PIVKA-II and PT-INR, mortality or SOFA score
Cheves et al. [115]	United States of America	N = 48 (15F, 33 M)	Prospective study	INR, procoagulant and anticoagulant clotting factors, anti-Xa	In 19/48 samples vitamin K deficiency pattern
					Decrease in plasma levels of vitamin K factors is a common cause of a high INR coagulopathy (with FVII as the primary driver of prolonged PT and elevated INR) at the ICU
		ICU patients with INR samples > 1.5 (20% of samples with INR > 1.5 used in this study)		Vitamin K deficiency pattern: fibrinogen normal or increased, FV level normal, and FVII, FX, PS and PC levels decreased (< 50%)	
Mulder et al. [83]	The Netherlands	N = 112 (232 patients in total cohort) (25F, 87 M)	Prospective cohort	dp-ucMGP concentration	Patients with COVID-19 show higher dp-ucMGP levels than the reference range at the time of intubation, and these levels remained stable during the ICU stay
		ICU patients with confirmed COVID-19 diagnosis (PCR + CT scan) and mechanical ventilation			After adjustment for confounders, significantly higher dp-ucMGP levels were found in ICU non-survivors compared to survivors
					Results suggest the role of dp-ucMGP, i.e. vitamin K shortness, as a marker for disease severity

dc-ucMGP: dephosphorylated-uncarboxylated matrix gla protein, ET: Echis time, ICU: Intensive Care Unit, PIVKA-II: protein induced by vitamin K absence-II, PT: prothrombin time, PT-INR: prothrombin time-international normalized ratio. N = number, F = female, M = male

**Table 2:** Studies evaluating vitamin K supplementation in critically ill patients

Authors and year of publication	Country of origin	Population and sample size	Methodology	Relevant outcome measures	Key findings
Alperin et al. [23]	USA	N = 42 (22F, 20 M)	Retrospective study 20-25 mg intravenous vitamin K1	PT, APTT, plasma levels of factors I, II, V, VII, VIII, IX and X	Improved coagulation after 4–6 h, complete correction within 12 h
					All patients were treated with two or more antibiotics
		Critically ill patients with prolonged PT and aPTT, normalized < 12 h of vitamin K1 supplementation			Recommend vitamin K prophylaxis for critically ill patients by giving 5 mg 2–3 × a week either orally or parenterally
Maclaren et al. [90]	Canada	N = 48 (19F, 29 M)	Retrospective observational study (monitoring prospectively)	INR values and aPTT	Two daily doses of intravenous vitamin K1 were associated with reduced INR values in patients with low to moderate disease severity, and the third dose was less effective
		Critically ill patients without consumptive coagulopathies or vitamin K therapy for pharmacologically induced INR prolongation or as a component of nutrition support	Daily doses of 10 mg of vitamin K1 intravenous		
					Daily aPTTs were unaffected by vitamin K1
					Vitamin K should only be administered if INR values are elevated, and INR should be used to monitor response
					No adverse events with intravenous vitamin K1
Dahlberg et al. [105]	Sweden	N = 258 (4,541 patients screened) (54F, 204 M)	Retrospective, single-center	PT-INR	Slightly more significant decrease of PT-INR 12–36 h after vitamin K supplementation compared to controls
			Intravenous vitamin K administration compared to the control group, doses ranged from 5 to 20 g		
		ICU patients with a PT between 1.3 and 1.9 (excluding liver cirrhosis)			
					No difference in mortality between vitamin K and the control group at 30, 90 and 180 days
Dahlberg et al. [24]	Sweden	N = 52 (16F, 36 M)	Prospective, single-center observational study	APTT, PT, fibrinogen, dp-ucMGP, PIVKA-II, FII, FVII, FIX, FX, protein C and S, vitamin K1 in nutrition	Vitamin K1 reduced PIVKA-II (n = 6) and dp-ucMGP (but not to normal levels)
					Vitamin K1 reduced PT time, but not aPTT time
			10 mg intravenous vitamin K1 (Samples ahead of treatment and after 24 h)		
					Increase in thrombin generation and coagulation factors II, VII, IX, and X, a slight/minor increase in protein C activity
		Critically ill, non-bleeding adult patients without liver failure or anticoagulation treatment, with an Owren PT > 1.2 and who were prescribed vitamin K1			
					Vitamin K1 initiated a predominantly pro-coagulative response
Sulaiman et al. [116]	Saudi Arabia	N = 98 (1,864 patients screened) (44F, 54 M)	Retrospective observational study	Incidence of bleeding INR	Only INR reduction after the first dose of vitamin K
		ICU patients with coagulopathy secondary to liver disease (INR > 1.5 within 24 h of ICU admission)			
				Mechanical ventilation duration, ICU length of stay, 30-day mortality	
			Vitamin K administration (via any route, 93.6% intravenous) with a median dose of 10 mg and for a median duration of three days, and control group		
					Patients who received vitamin K for INR correction have longer ICU LOS and mechanical ventilation duration
					No differences in the odds of a new bleeding event or thrombosis
					Patients who received vitamin K were 2.4 times more likely to get VTE (*p* = 0.3)
Schött et al. [126]	Sweden	N = 52 (16F, 36 M)	Prospective screening study (substudy of [24])	Gas6 and sAxl plasma concentrations	Significant but slight increase in median Gas6 over 20–28 h, but not in the median level of sAxl receptor
		Non-warfarin-treated ICU patients with PT-INR > 1.2	10 mg intravenous vitamin K supplementation		
Gudivada et al. [42]	India	N = 65 (23F, 42 M)	Prospective observational study	INR elevation > 1.5 in 6-day study period	17 (26%) patients had elevated INR during the 6-day study period
			Prophylactic (N = 22) or therapeutic (N = 11) vitamin K administration (route not specified, dose ranging from 10–30 mg for 1–3 days)		No protection of prophylactic vitamin K use against elevation of INR
		ICU patients administered cefoperazone (not on warfarin therapy, pre-existing elevated PT/INR, or vitamin K1 therapy)			
					11/17 patients with elevated INR received therapeutic vitamin K, and 10/11 had a significant decrease in INR to the normal range

dc-ucMGP: dephosphorylated-uncarboxylated matrix gla protein, ICU: Intensive Care Unit, LOS: length of stay, PIVKA-II: protein induced by vitamin K absence-II, PT: prothrombin time, PT-INR: prothrombin time-international normalized ratio, VTE: venous thromboembolism. N = number, F = female, M = male

### Vitamin K deficiency risk factors in ICU patients

Despite limited knowledge of vitamin K deficiency in ICU patients, critical illness poses a potential risk. Vitamin K deficiency may occur upon ICU admission and could exacerbate during ICU stay [14, 15]. Dahlberg et al. [15] demonstrated that ICU patients exhibit higher PIVKA-II values upon admission than healthy adults, indicating a decreased vitamin K status. Moreover, during ICU stay, these PIVKA-II values significantly increased. This increase may occur rapidly due to lower circulating levels and tissue storage of vitamin K compared to other fat-soluble vitamins [9]. Critically ill kidney failure patients may have lower vitamin K levels due to impaired recycling from uremia, dietary restrictions, and increased utilization of vitamin K-dependent proteins to prevent calcification [18–20]. Critically ill patients without known risk factors for vitamin K deficiency may also develop vitamin K deficiency [16]. Several ICU-related factors may contribute to this deficiency, including inadequate vitamin K supply, malabsorption, antibiotic treatment, increased physiological vitamin K turnover, greater need, and disrupted vitamin K recycling.

#### Malnutrition and impaired absorption of vitamin K

Malnutrition, highly prevalent among ICU patients, rapidly impacts their vitamin K status [21–23]. Body stores of vitamin K are limited, necessitating frequent intake to maintain adequate status and recycling. Critically ill patients often receive less than the recommended daily amount of vitamin K, as enteral and parenteral nutrition may not consistently provide reference levels of this vitamin [24–26]. However, low plasma vitamin K levels are not solely attributed to protein-energy malnutrition in hospitalized patients [27]. Vitamin K is absorbed in the small intestine. Factors contributing to impaired absorption in ICU patients include intestinal inflammation and gastric retention [24].

Additional factors for vitamin K deficiency are extrahepatic biliary obstruction and severe pancreatic insufficiency. These conditions may affect vitamin K absorption, as it relies on being incorporated into mixed micelles of bile salts and pancreatic lipolysis products [28]. Moreover, disturbed lipid homeostasis during critical illness may impact the transport and absorption of vitamin K, which is dependent on lipoproteins [9, 29]. ICU patients' microbiome disruption worsens with the use of gastric acid inhibitors, vasoactive agents, and opioids, as well as total parenteral or enteral nutrition with processed liquid diets [30–32]. Vitamin K absorption relies on the Niemann-Pick C1-like 1 (NPC1L1) transporter in the intestine. Concurrent use of vitamin E supplementation or ezetimibe (used for dyslipidemia), which also utilizes the NPC1L1 transporter, may reduce vitamin K absorption [33, 34].

#### Antibiotics

Antibiotics, prescribed to up to 70% of ICU patients, can worsen vitamin K deficiency by disturbing gut bacteria essential for its synthesis through microbiome disturbance, thereby potentially reducing vitamin K absorption in the intestines [35–37]. Certain antibiotics containing an N-methylthiotetrazole group, such as second and third-generation cephalosporins, have been found to hinder the synthesis of coagulation factors. This inhibition occurs by blocking vitamin K epoxide reductase, disrupting gamma-carboxylation, which mirrors the mechanism observed with vitamin K epoxy reductase inhibitors [38–42]. The clinical impact of antibiotics is pronounced in patients with a low vitamin K status [38].

#### Increased vitamin K turnover

Critically ill patients face increased demand for vitamin K alongside inadequate supply. Factors contributing to this increased demand include metabolic stress, inflammation, oxidative stress, and organ dysfunction. Moreover, critically ill patients may undergo heightened degradation of factor VII, potentially exacerbating an underlying vitamin K deficiency [39]. Vitamin K supply–demand imbalance becomes especially concerning with inadequate recycling. Vitamin K epoxide reductase (VKOR), an enzyme crucial for converting oxidized vitamin K epoxide into its active reduced hydroquinone form, has garnered attention. Vitamin K antagonists exert their effect by inhibiting the enzymatic activity of VKOR and vitamin K reductase (VKR), hindering the conversion of vitamin K to its active form. ICU patients with hypoalbuminemia may experience unstable vitamin K levels when exposed to vitamin K antagonists, as these drugs primarily bind to albumin in the body [43].

#### Genetic polymorphisms

Variability in vitamin K levels in ICU patients may be linked to genetic polymorphisms. Specific polymorphisms in the apolipoprotein E gene impact vitamin K uptake. This lipoprotein facilitates the uptake of vitamin K in tissues by binding to low-density lipoprotein to the low-density lipoprotein receptor [9, 44]. Additionally, genetic polymorphisms may contribute to the variable response to vitamin K antagonists, including those associated with VKORC1. However, a minor role has been observed for GGCX polymorphisms and cytochrome P450 (CYP) enzymes, namely CYP2C9 and CYP2C19 [45–47]. When using vitamin K antagonists with VKORC1 polymorphisms, dose adjustment may be necessary to reduce the bleeding risk [11, 48]. Additionally, various drugs, including cocaine, can inhibit CYP2C9 enzyme activity, potentially affecting vitamin K function [45, 49].

### Impact of vitamin K deficiency on critically ill patients

Vitamin K deficiency poses significant consequences for ICU patients. However, defining it proves challenging due to the need for more straightforward diagnostic tests. Elevated PIVKA levels often indicate subclinical deficiency, while clinical deficiency manifests when coagulation is affected. Given vitamin K's pivotal role in clotting factor production, deficiency can lead to hypoprothrombinemia, heightening the risk of bleeding. Although rare, cases of gastrointestinal bleeding associated with vitamin K deficiency have been reported in the literature [41, 50]. Hepatic insufficiency, impacting approximately 15% of ICU patients, worsens clotting factor deficiency, heightening bleeding risk [39].

#### Microvascular bleeding in the lung

Vitamin K deficiency increases the risk of microvascular bleeding or diffuse alveolar hemorrhage (DAH) in ICU patients. This bleeding, involving blood accumulation in lung tissue, has been linked to vitamin K deficiency caused by coumarin anticoagulants [51–53]. Iron accumulation during DAH causes oxidative stress and inflammation, contributing to the pathophysiology of fibrotic lung diseases [45, 47, 54–57]. In severe cases, DAH may progress to respiratory failure, necessitating mechanical ventilation and higher mortality rates. This may have significant implications for ICU patients, especially those with genetic polymorphisms associated with impaired vitamin K regeneration.

#### Procoagulant state

The triage theory proposes that depleted vitamin K stores prioritize hepatic carboxylation, leading to a significant procoagulant imbalance [58]. Critically ill patients have lower protein C levels and possibly a shift from free to bound protein S, promoting a more prothrombotic state and no longer preventing local thrombosis in the vessel wall [58–60]. This becomes self-perpetuating as heightened hypercoagulability increases clotting factor consumption, further depleting vitamin K stores [61]. Possible reasons for reduced protein S and C levels in critically ill patients include increased consumption, reduced production, and excessive extravasation due to increased vascular permeability [61]. Sepsis-induced coagulopathy has been observed, partially due to suppressed protein C levels and association with microvascular coagulation, organ dysfunction, and increased mortality [62–64]. It has also been observed that patients with ventilator-acquired pneumonia experience a decline in pulmonary protein C levels prior to its onset [60, 63]. This may be even more profound in the presence of vitamin K deficiency.

#### Elastic fiber degradation

Since hepatic carboxylation is favored in vitamin K deficiency, the influence extends to other proteins beyond the liver. Insufficient pulmonary MGP carboxylation hampers vascular calcification control, potentially causing pulmonary emphysema and decreased lung function [58, 65, 66]. High dp-ucMGP levels, an indicator of inactive MGP, have been linked to accelerated elastic fiber degradation [67]. High dp-ucMGP levels have also been observed in hospitalized COVID-19 patients, associated with a higher risk of invasive ventilation or mortality [67]. This can be explained by COVID-19 virus proteolytic activity, generating matrix metalloproteinases with the degradation of elastic fibers and increased calcium accumulation, stimulating MGP synthesis and depleting extrahepatic vitamin K stores [58]. The results of a recently conducted randomised, placebo-controlled trial investigating the effects of vitamin K2 supplementation in patients hospitalised with COVID-19 have demonstrated that such supplementation has the potential to reduce levels of dp-ucMGP and PIVKA-II without increased risk of thrombosis [68]. Elevated matrix metalloproteinase levels in ICU patients [69] may increase vitamin K consumption for MGP carboxylation. Increased uncarboxylated MGP is also associated with long-term arterial stiffness, vascular and valvular calcification, heart failure, and increased cardiovascular mortality [70].

#### Other extrahepatic functions

A recently discovered mechanism reveals that vitamin K plays a role in suppressing ferroptosis through the ferroptosis suppressor protein 1 (FSP1), functioning as a vitamin K reductase. FSP1 converts vitamin K back into its corresponding hydroquinone, also acting as a potent radical-trapping antioxidant. Consequently, it protects cells against ferroptosis and lipid peroxidation [71]. During oxidative stress, VKORC1L1 and VKOR are upregulated, and VKORC1L1 may drive vitamin K-mediated intracellular antioxidation, which is crucial for cell survival [72]. Vitamin K may also suppress and prevent vascular inflammation and insulin resistance in type-2 diabetes [73–75]. Furthermore, vitamin K plays a pivotal role in the maintenance of bone health, which is of particular significance during the post-intensive care period, characterised by accelerated bone loss and an elevated risk of fragility fractures [76].

#### Role in inflammation

Vitamin K exerts a beneficial influence on the course of infections, inflammation and autoimmune diseases, particularly through its anti-inflammatory and antioxidant properties [77]. Furthermore, vitamin K is involved in the carboxylation of Gas6, which has been identified as a potential inflammation marker in ICU patients. Elevated levels of Gas6 have been observed in patients with severe sepsis [78–82]. Although soluble AxL levels increase concurrently, believed to inhibit Gas6 activity by binding with it, the upregulation of Gas6 during sepsis exceeds that of sAxl [78]. Gas6 potentially possesses antiapoptotic and pro-survival properties while regulating the inflammatory response in hyperinflammatory states [79]. Furthermore, Gas6 may attenuate neutrophil infiltration into the lungs during sepsis, which is crucial in acute lung injury [79]. Recent COVID-19 studies propose dp-ucMGP as a potential severity marker. In both hospitalized and severely ill COVID-19 patients, dp-ucMGP levels were high [67, 83]. Specifically, ICU non-survivors exhibited higher dp-ucMGP levels over time [67, 83].

### Vitamin K supplementation

ICU studies on vitamin K supplementation reveal improved status but incomplete plasma normalization (Table 2). Randomized controlled studies are pending. Critically ill patients may respond inadequately to vitamin K supplementation, and the ideal dosage and administration route remain uncertain [39, 84].

#### Route of administration

ICU patients may lack sufficient vitamin K, especially if they develop or sustain a deficiency during their ICU stay [14, 15]. Vitamin K can be administered orally, intravenously, subcutaneously, or intramuscularly. Absorption depends on bile salts, the presence of other lipids, and pancreatic enzymes when taken orally [28]. Patients with excessive anticoagulation face a heightened risk of hematoma formation with intramuscular or subcutaneous administration [39]. Additionally, subcutaneous administration may be unpredictable and less effective [85, 86]. Intravenous administration may cause hemodynamic instability and anaphylactic reactions. Therefore, a slow infusion rate should be maintained [87, 88]. Studies indicate oral vitamin K supplements result in a slower reduction in INR ratio, while the speed of INR reduction with intravenous supplementation is dose-dependent [89].

#### Type of administration

Optimal vitamin K supplementation dosage and timing must be established due to varying synthesis rates of vitamin K-dependent coagulation factors [24]. Repeated intravenous doses of 10 mg of vitamin K were given to ICU patients, with the most significant change after two consecutive doses [90]. A comprehensive review suggests supplementation of at least 1 mg orally daily or 10 mg intravenously weekly in ICU patients but also emphasizes the need for further research [91]. In over-anticoagulation cases, intravenous and oral vitamin K can correct the INR within 24 h [92]. A 10 mg intravenous vitamin K1 dose after 24 h reduced uncarboxylated extrahepatic Gla proteins but did not normalize them [24]. Similarly, in critically ill children with prolonged antibiotic use, no change in vitamin K deficiency was observed after a single weight-dependent dose (0.5 mg/kg, maximum 10 mg) of vitamin K [93]. Additionally, older ICU patients may require higher and prolonged doses of vitamin K [94]. Observational studies propose vitamin K2's superior cardiovascular protection over K1 [95, 96]. However, therapeutic trials have also demonstrated similar effects with vitamin K1 [97]. Recent animal research has demonstrated that vitamin K2 is a potential therapeutic option for acute lung injury. It may alleviate acute lung injury by regulating inflammation, apoptosis, ferroptosis, and elastin degradation [98]. This may be due to the longer half-life of vitamin K2 [66].

#### Vitamin K in the non-bleeding critically ill patient

While commonly used to correct elevated INR preoperatively, vitamin K is not routinely recommended for non-bleeding critically ill patients [99]. In some cases, intravenous vitamin K supplementation is administered to patients with prolonged prothrombin complex (PT-INR) time [100], even without using vitamin antagonists or non-liver failure. Intravenous supplementation of vitamin K1 can shorten PT time and increase vitamin K-dependent coagulation factor activity and thrombin formation [24]. In normal physiological conditions, vitamin K supplementation primarily targets the enhancement of extrahepatic carboxylation of vitamin K-dependent proteins [101–103]. VKOR activity is three times higher in vascular smooth muscle cells than in hepatocytes [104], possibly because vitamin K is transported to the liver very efficiently. This may elucidate the limited impact of vitamin K supplementation on PT-INR values observed in ICU patients [105] and explain the presence of non-carboxylated forms of MGP and OC in non-supplemented adults [7, 106, 107].

#### Vitamin K antagonists

The role of vitamin K in counteracting the excessive anticoagulant effect of vitamin K antagonists is well established [108, 109]. Vitamin K supplementation may reduce day-to-day variability in vitamin K intake, resulting in a more stable INR and reduced bleeding complications [55, 110, 111]. Vitamin K supplementation should be given with careful INR monitoring to avoid INR dropping below therapeutic levels, which may heighten thromboembolic risk [112]. Conversely, vitamin K supplementation may boost antithrombotic activity, frequently diminished in those with vitamin K deficiency [55]. Since vitamin K exhibits procoagulant and anticoagulant properties, the balance between these effects can be influenced by the subtype-specific vitamin K administered [113]. Vitamin K1 may show a particularly pro-coagulative response, with the pro-coagulative proteins increasing more than proteins C and S [24].

## Discussion

Risk factors for vitamin K deficiency in ICU patients include inadequate supply, malabsorption, antibiotic use, increased physiological consumption, and impaired recycling. This scoping review is the first to explore the mechanisms and consequences of vitamin K deficiency in this population. Six studies analyzing ICU patients' vitamin K status indicate many patients may have (sub)clinical deficiency upon admission and during their stay [14–16, 83, 114, 115]. Deficiency may appear subclinical with normal PT but high PIVKA levels, possibly masking extrahepatic vitamin K function due to hepatic carboxylation favoring [15]. Consequently, microvascular coagulation, organ dysfunction, and an increased mortality risk can occur [62–64]. Vitamin K deficiency in the ICU setting can exacerbate during admission, potentially compromising hepatic functions and impairing coagulation factor production. This may cause bleeding events at various levels, including DAH, triggering inflammation and possibly leading to respiratory failure requiring mechanical ventilation and adverse outcomes. Moreover, beyond short-term consequences, ICU patients may endure long-term effects of vitamin K deficiency, such as vascular calcification in the lungs and cardiovascular system [58, 65, 70]. The impact of vitamin K supplementation on clinical outcomes has been sparsely studied. In an observational study by Dahlberg et al., no significant difference was observed in mortality rates at 30, 90, and 180 days of vitamin K supplementation (100). Sulaiman et al. found that vitamin K supplementation was associated with an elevated risk of thrombosis, prolonged ICU stay, and mechanical ventilation. Importantly, this was observed only in critically ill patients with liver disease and prolonged INR levels [116].

### Evaluation of vitamin K status in ICU patients

**Fig. 4:**
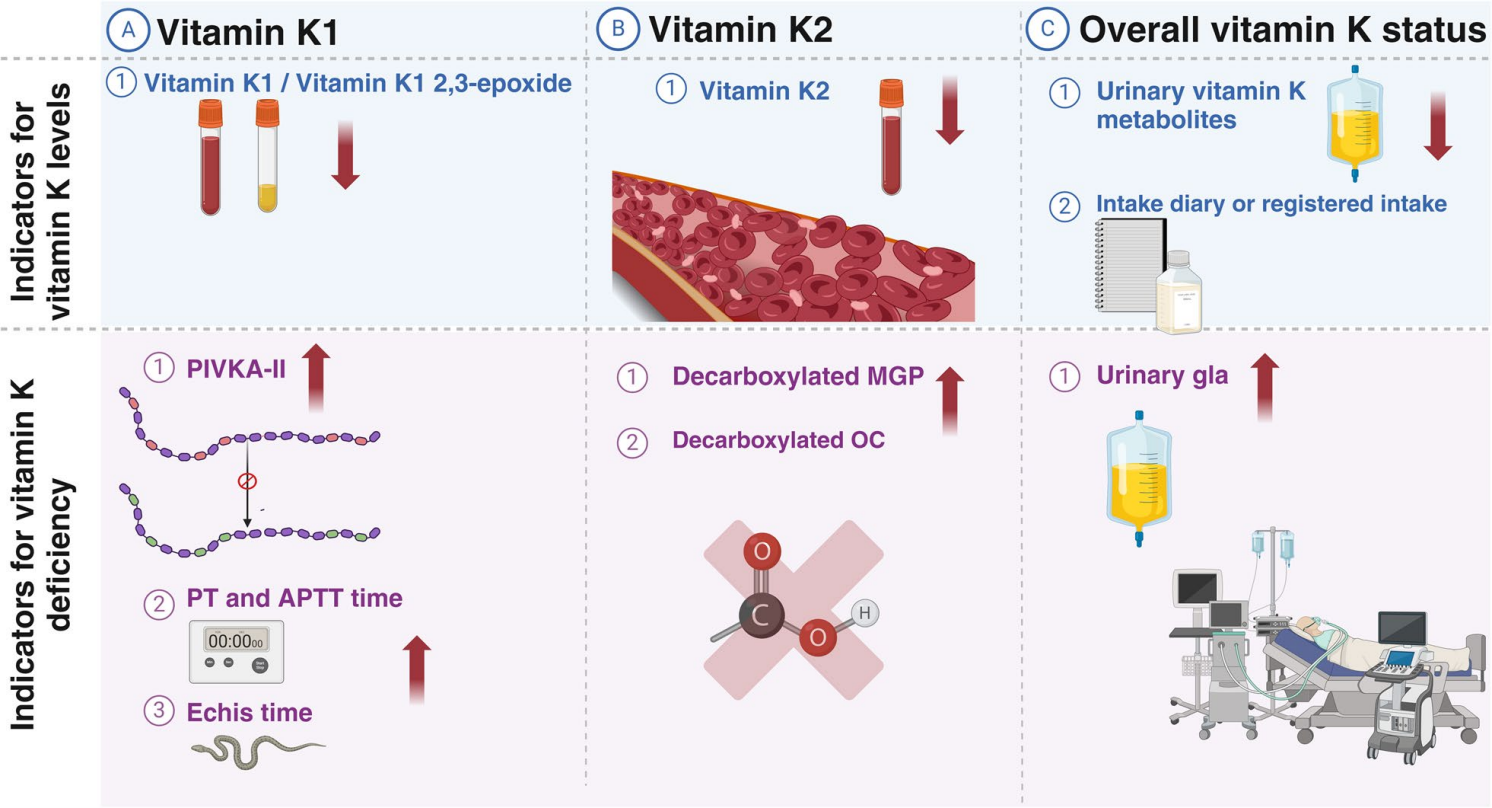
Assessment of Vitamin K status. Figure 4 Red arrows indicate changes in measurements due to vitamin K deficiency. Quantifying vitamin K status is challenging due to various dietary intakes and the complexity of detecting vitamin K2 without supplementation [84]. Measurement accuracy may require adjustments and fasting samples, as vitamin K circulates with triglyceride-rich lipoproteins [84, 114]. Assessment of hepatic vitamin K status commonly relies on prothrombin time (PT) and PT-internal normalized ratio (PT-INR), but PT lacks sensitivity, particularly in the presence of liver dysfunction or hematological diseases. Furthermore, PT offers restricted insights as it exclusively concentrates on procoagulants while neglecting anticoagulants and extrahepatic functions. Consequently, it provides an incomplete reflection of overall vitamin K status [117]. Uncarboxylated gla proteins, such as uncarboxylated factor II (PIVKA-II), desphospho-uncarboxylated MGP (dp-uc MGP), and uncarboxylated osteocalcin (ucOC) are gaining attention for assessing extrahepatic vitamin K use. However, elevated dp-uc MGP and ucOC levels do not always indicate suboptimal carboxylation of hepatic proteins [106, 118]. In critical illness, PIVKA-II levels rise, possibly due to the acute phase response, complicating interpretation [15]. Echis time, using viper venom, provides an alternative method to assess vitamin K status. Echis time uses viper venom (Echis carinatus) to activate normal prothrombin and PIVKA-II to form thrombin. Consequently, the Echis time remains within the normal range in the presence of vitamin K deficiency and is only prolonged in the presence of inadequate clotting factor production. However, its applicability in other critically ill patients requires further validation [119]. Urinary biomarkers such as y-carboxyglutamic acid (gla) reflect overall vitamin K-dependent protein status but have limitations, such as the need for 24-h urine samples, lack of correlation with dietary intake, and dependence on lean body mass. Created with BioRender.com

Various biomarkers exist for assessing vitamin K status, each with unique strengths, limitations, and interpretation challenges. However, most studies utilizing these biomarkers have focused on healthy volunteers. Various laboratory methods measure phylloquinone and menaquinones in human blood. Biomarkers such as the ratio or percentage of un- or under-carboxylated osteocalcin, MGP, and PIVKA-II have been proposed to reflect vitamin K status and/or storage, such as in liver or bone tissue. Traditional indicators like PT, PTT, and coagulation factors are relatively insensitive and nonspecific for assessing vitamin K status. Given vitamin K's hepatic and extrahepatic effects, comprehensive markers covering both functions are needed for accurate assessment. Independent assessments are available to distinguish between the various roles of vitamin K (Fig. 4).

Furthermore, the complexity of assessing vitamin K levels is compounded by differences in the bioavailability and half-life of vitamin K1 and K2 and the conversion of phylloquinone into menaquinone-4 in the gastrointestinal tract [67]. Inter- and intra-individual variation in plasma K levels further complicates interpreting whether hepatic stores are being reflected [84]. Elevated levels of uncarboxylated osteocalcin and matrix Gla protein may not necessarily indicate suboptimal carboxylation of hepatic vitamin K-dependent proteins or inadequate vitamin K availability. Moreover, optimal carboxylation levels have yet to be defined. This could be due to prioritizing hepatic γ-carboxylation upon vitamin K intake [67, 103, 118]. The clinical relevance of elevated PIVKA-II levels in ICU patients remains unclear, and whether they indicate subclinical deficiency is uncertain. Elevated PIVKA-II levels alongside normal PT-INR have been observed in general ICU patients and those with sepsis [15, 120]. It has been proposed that this phenomenon is linked to the acute phase response in ICU patients. PIVKA-II production may occur during catabolic periods despite sufficient vitamin K levels due to an imbalance between protein supply and post-translational carboxylation capacity [15, 114, 121]. To ascertain the clinical significance of elevated levels of uncarboxylated proteins, Further investigation is warranted considering the time lag for reduced plasma levels to affect hepatic reserves and function [114]. Gas6 may not be a reliable indicator of vitamin K deficiency, but potentially a marker of disease severity in sepsis patients. The metabolic responses of critically ill patients to disease, injury, and infection can independently affect laboratory results, irrespective of dietary intake and nutritional status. The presence of inflammation, which is commonly observed in these conditions, introduces a degree of complexity when interpreting blood levels [26, 122].

### Strengths and limitations

This scoping review represents the first exploration of vitamin K's role in critical illness. Several limitations were encountered, including a need for more available observational studies and the absence of randomized controlled trials. Complexity in study comparison hindered meta-analysis due to the absence of validated tests for detecting and treating vitamin K deficiency. The lack of established reference values for vitamin K deficiency in critically ill ICU patients also complicated comparisons. Differentiating between clinical and subclinical vitamin K deficiency was challenging due to limited studies investigating their effect on key clinical outcomes.

## Summary

**Fig. 5:**
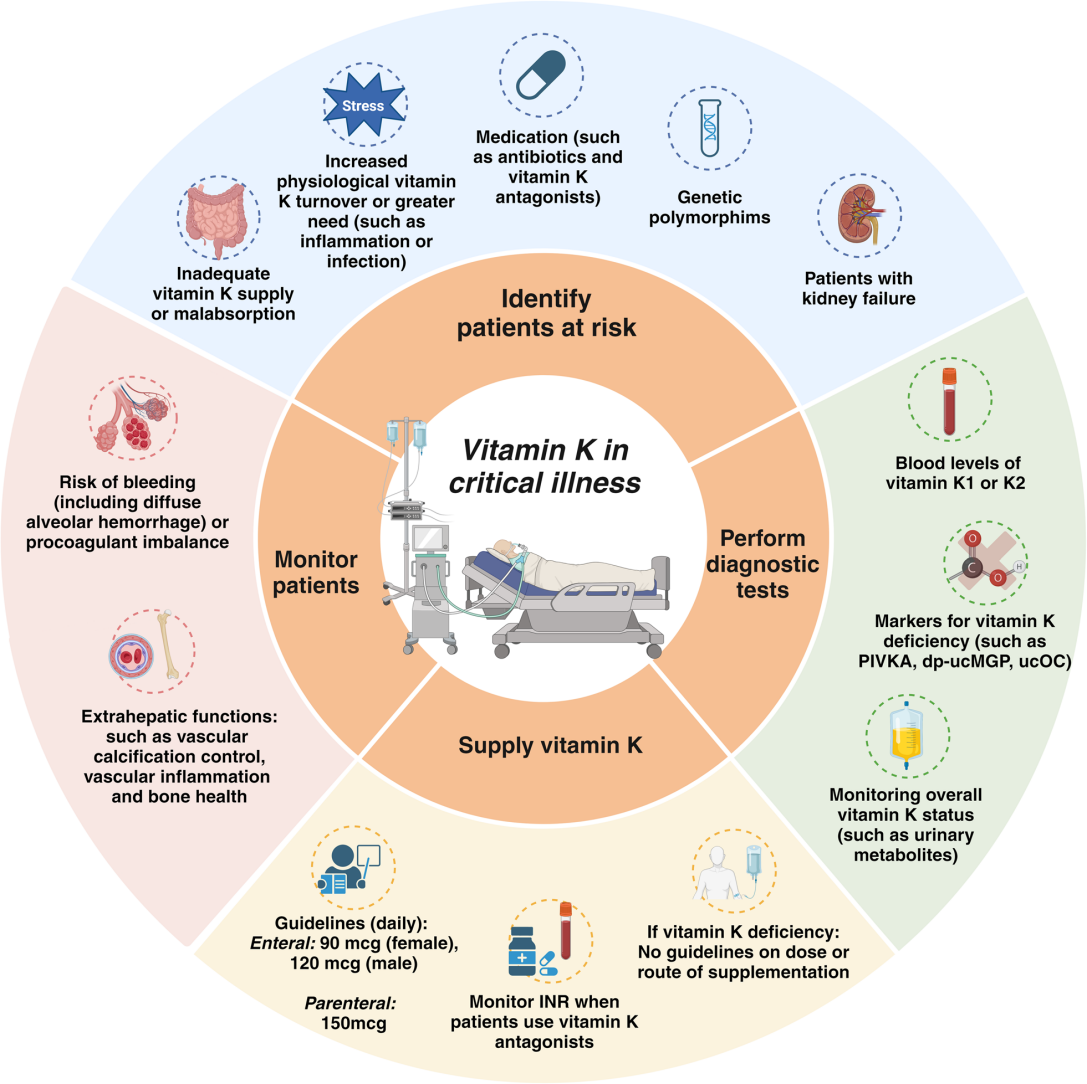
Practical tool to manage Vitamin K for critically ill patients in daily clinical practice. This figure provides an overview of the management of vitamin K during critical illness. Critically ill patients are known to have a high prevalence of vitamin K deficiency, which can worsen during ICU admission. Patient categories at risk are listed. The complexity of assessing vitamin K levels is compounded by the availability of various diagnostic tests. So far, no consensus on the optimal method to diagnose vitamin K deficiency in critically ill patients exists. International guidelines have been developed for the minimum daily intake of vitamin K in critically ill patients. However, uncertainty remains regarding the optimal dosage and route of administration to correct vitamin K deficiency. Furthermore, the figure emphasises the importance of monitoring patients at risk of vitamin K deficiency. Abbreviations: PIVKA: proteins induced by vitamin K absence or antagonist, dp-ucMGP: dephospho-uncarboxylated matrix Gla protein, ucOC: undercarboxylated osteocalcin. Created with BioRender.com

In summary, our study underscores the prevalence and progression of vitamin K deficiency in critically ill patients, highlighting their potential risk for impaired coagulation function, prolonged mechanical ventilation, and increased mortality (Fig. 5). However, there is still a lack of knowledge regarding the analysis methodology of vitamin K levels; this is mainly due to the absence of consensus on the use of specialized tests for assessing vitamin K status in this population and the underestimated role of vitamin K in critical illness. It is essential to recognize that even without abnormal values of vitamin K1, the extrahepatic functions of vitamin K may already be compromised [84].

Assessing PIVKA-II, reflecting subclinical vitamin K deficiency, might be an option, although its reliability is contingent on the patient not being in a catabolic state. Pre-emptive mapping of a pharmacogenetic profile in critically ill patients, particularly those with multidrug use, with or without anticoagulants, may be beneficial and worthy of consideration. This approach appeared helpful in elective ICU admissions [123]. However, further studies are needed to explore the benefits for urgently admitted patients.

Critically ill patients necessitate substantial enteral or parenteral nutrition to meet the vitamin K requirements outlined by organizations such as the European Society for Clinical Nutrition and Metabolism (120 mcg per 1500 kcal of enteral feed and 150 mcg with parenteral nutrition) and the American Society for Parenteral and Enteral Nutrition (120 mcg for men and 90 mcg for women daily and 150 mg with parenteral nutrition) [25, 26]. It is important to note that patients may receive vitamin K from intravenous lipids, which are frequently used for parenteral nutritional (PN) support. Intravenous lipids are administered concomitantly with continuous sedation in patients receiving propofol, which contains 10% soybean oil as an emulsified preparation. It is notable that soybean oil represents a significant source of vitamin K [124, 125]. Additionally, critically ill patients have diminished vitamin K absorption capabilities and potentially increased demand, rendering them vulnerable to deficiency. Personalized nutrition may be imperative for patients with VKORC1 polymorphisms or those with excessive vitamin K consumption. Adequate administration of multivitamins containing vitamin K or intermittent vitamin K supplementation to mitigate deficiency is recommended. So far, the optimal dosage for critically ill patients is lacking, mainly because supplementation studies have indicated that vitamin K levels did not normalize despite supplementation [24, 105, 116]. A minimum of 1 mg orally per day or 10 mg weekly could be considered [23, 91].

Patients taking antibiotics, with prolonged ICU stays, or at risk of lung damage may require increased vitamin supplementation. Also, it may be prudent to consider continuing vitamin K supplementation even after discharge from the ICU to mitigate the potential long-term effects of vitamin K deficiency. Although no studies have investigated the long-term effects of vitamin K deficiency in critically ill patients, vitamin K suppletion is safe. It does not increase the risk of thrombotic events [7]. However, monitoring is vital when vitamin K antagonists are used, ensuring an INR between therapeutic ranges. Whether vitamin K supplementation may improve the vitamin K status of critically ill patients and, in turn, influence ventilator duration and mortality outcomes warrants future research. Moreover, further research is necessary to identify at-risk groups and determine personalized vitamin K dosages.

### Supplementary Information

Supplementary material 1.

## Data Availability

Data sharing is not applicable to this article as no datasets were generated or analyzed during the current study. The search strategy is included in the online data supplement.

## Abbreviations

CYP: Cytochrome
DAH: Diffuse alveolar hemorrhage
Dp-ucMGP: Dephosphorylated, uncarboxylated MGP
FSP1: Ferroptosis suppressor protein 1
Gas6: Growth arrest sequence-6 protein
GGCX: Gamma-glutamyl carboxylase
Gla: Gamma-carbo-glutamic acid
ICU: Intensive care unit
MGP: Matrix Gla protein
MK: Menaquinone
NPC1L1: Niemann-Pick C1-like 1 (NPC1L1)
OC: Osteocalcin
PIVKA: Proteins induced in vitamin K absence or antagonism
PT: Prothrombin time
PT-INR: Prothrombin time-international normalized ratio (PT-INR)
SR-BI: Scavenger receptor class B-type I
ucOC: Uncarboxylated osteocalcin
VKOR: Vitamin K epoxide reductase
VKR: Vitamin K reductase
