# Supplementary material for: Vitamin K: a potential missing link in critical illness–a scoping review
Source: Crit Care. 2024 Jul 1;28:212. doi: 10.1186/s13054-024-05001-2 (PMC11218309; doi:10.1186/s13054-024-05001-2)
Supplement: Supplementary file 1 — Supplementary material 1. [file 13054_2024_5001_MOESM1_ESM.docx]

**Online Data Supplement**

**A scoping review on Vitamin K: a potential missing link in critical illness**

**Michelle Carmen Paulus MD, Marjolein Drent, MD PhD, Imre Willemijn Kehinde Kouw, RD PhD, Michiel Gerard Juliaan Balvers, Aalt Bast, PhD, & Arthur Raymond Hubert van Zanten, MD PhD**

| **Search terms** | **Results**  **[December 20th, 2023]** |
| --- | --- |
| **Pubmed** | |
| "Vitamin K"[Title/Abstract] OR "Vitamin K"[Mesh] OR "Vitamin K Deficiency"[Title/Abstract] OR "Vitamin K Deficiency"[Mesh] | 31,112 |
| OR |  |
| "matrix Gla protein" [Supplementary Concept] OR "gla protein"[Title/Abstract] OR "gla-protein"[Title/Abstract] OR "osteocalcin"[Title/Abstract] OR "Osteocalcin"[Mesh] OR "MGP"[Title/Abstract] OR "Mglap"[Title/Abstract] OR "Gas6"[Title/Abstract] | 24,338 |
| OR | |
| "PIVKA-II" [Title/Abstract] OR "PIVKA-2"[Title/Abstract] OR "PIVKA"[Title/Abstract] OR "protein induced by vitamin K absence" [Title/Abstract] OR "protein induced by vitamin K" [Title/Abstract] OR "acarboxy prothrombin"[Title/Abstract] OR "des-gamma carboxyprothrombin"[Title/Abstract] OR "acarboxyprothrombin" [Supplementary Concept] OR "dp-ucMGP"[Title/Abstract] | 1,689 |
| AND | |
| "intensive care units"[MeSH Terms] OR "Intensive Care"[Title/Abstract] OR "ICU"[Title/Abstract] OR "critical care"[MeSH Terms] OR "critical care"[Title/Abstract] OR "critically ill"[Title/Abstract] OR "critical illness"[Title/Abstract] | 336,889 |
| **Combined** | 310 |
| **Embase** | |
| **'vitamin k'/exp OR 'vitamin k'**:ti,ab,kw **OR 'vitamin k deficiency'**:ti,ab,kw **OR 'vitamin k deficiency'/exp** | 53,918 |
| OR | |
| **‘gla protein’**:ti,ab,kw **OR ‘gla-protein’**:ti,ab,kw **OR ‘osteocalcin’/exp OR ‘osteocalcin’**:ti,ab,kw OR ‘MGP’:ti,ab,kw OR ‘mglap’:ti,ab,kw OR ‘growth arrest specific protein 6’/exp OR ‘growth arrest specific protein 6’:ti,ab,kw OR ‘gas6’:ti,ab,kw | 39,975 |
| OR | |
| ‘PIVKA’:ti,ab,kw OR ‘PIVKA-II’:ti,ab,kw OR ‘PIVKA-2’:ti,ab,kw OR ‘protein induced by vitamin K absence’:ti,ab,kw OR ‘protein induced by vitamin K’:ti,ab,kw OR ‘descarboxyprothrombin’/exp OR ‘descarboxyprothrombin’:ti,ab,kw OR ‘ascorby prothrombin’:ti,ab,kw OR ‘des-gamma carboxyprothrombin’:ti,ab,kw OR ‘ascarboxyprothrombin’:ti,ab,kw OR ‘dp-ucMGP’:ti,ab,kw | 3,561 |
| AND | |
| ‘intensive care’:ti,ab,kw OR ‘ICU’:ti,ab,kw OR ‘intensive care unit’/exp OR ‘critical care’:ti,ab,kw OR ‘critically ill’:ti,ab,kw OR ‘critically ill patient’:ti,ab,kw OR ‘critical illness’:ti,ab,kw OR ‘critical ilness’/exp | 561,014 |
| **Combined** | 1521 |
| **Cochrane** | |
| "Vitamin K" OR "Vitamin K Deficiency" OR "Vitamin K Deficiency" OR "matrix Gla protein" OR "gla protein" OR "gla-protein" OR "osteocalcin" OR "MGP" OR "Mglap" OR "Gas6" OR "PIVKA-II" OR "PIVKA-2" OR "PIVKA" OR "protein induced by vitamin K absence" OR "protein induced by vitamin K" OR "acarboxy prothrombin" OR "des-gamma carboxyprothrombin" OR "acarboxyprothrombin" OR "dp-ucMGP" | 169 |
| AND | |
| "intensive care units" OR "Intensive Care" OR "ICU" OR "critical care" OR "critical care" OR "critically ill" OR "critical illness" | 1746 |
| **Combined** | 34 |

**Supplementary Table 1: Search Strategy**

**Supplementary Figure 1: PRISMA flow chart of included studies**


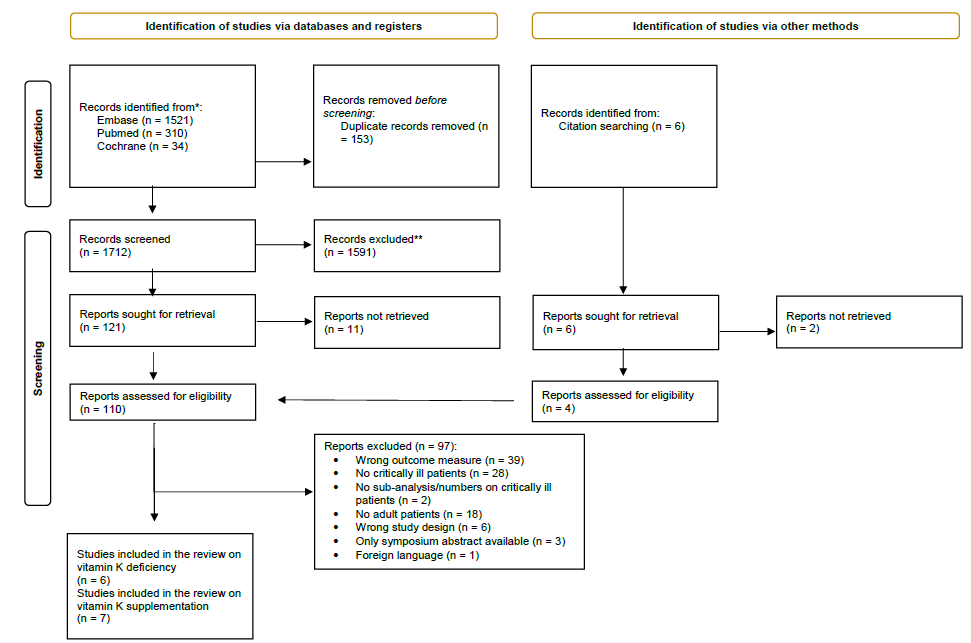


**Supplementary Table 2: JBI Critical Appraisal Table**

|  | **JBI Critical Appraisal Checklist for Cross-sectional studies** | | | | | | | | | | | |
| --- | --- | --- | --- | --- | --- | --- | --- | --- | --- | --- | --- | --- |
| **Author(s)** | **Q1** | **Q2** | **Q3** | **Q4** | **Q5** | **Q6** | **Q7** | **Q8** | **Q9** | **Q10** | **Q11** | **Total** |
| Alperin et al. [19] | N | N | Y | Y | U | U | Y | N |  |  |  | 3/8 |
| Chakraverty et al. [11] | Y | Y | Y | Y | Y | U | Y | Y |  |  |  | 7/8 |
| Cheves et al. [109] | N | Y | Y | Y | U | U | Y | Y |  |  |  | 5/8 |
| Crowhter et al. [9] | N | Y | Y | Y | U | U | Y | Y |  |  |  | 5/8 |
| Dahlberg et al. [10] | Y | Y | Y | Y | Y | Y | Y | Y |  |  |  | 8/8 |
| Dahlberg et al. [20] | Y | Y | Y | Y | Y | Y | Y | Y |  |  |  | 8/8 |
| Gudivada et al. [38] | Y | Y | Y | Y | Y | Y | Y | Y |  |  |  | 8/8 |
| Mulder et al. [77] | Y | Y | Y | Y | Y | Y | Y | Y |  |  |  | 8/8 |
| O’Shaugnessy et al. [108] | Y | Y | Y | Y | U | U | Y | Y |  |  |  | 6/8 |
| Schött et al. [120] | Y | Y | Y | Y | U | U | Y | Y |  |  |  | 6/8 |
|  | **JBI Critical Appraisal Checklist for Cohort Studies** | | | | | | | | | | | |
| Maclaren et al. [84] | N | N | Y | Y | Y | N | Y | Y | Y | N | Y | 7/11 |
| Dahlberg et al. [99] | Y | Y | Y | Y | Y | N | Y | Y | U | U | Y | 9/11 |
| Sulaiman et al. [110] | Y | Y | Y | Y | Y | Y | Y | Y | U | U | Y | 9/11 |

Appropriate appraisal for either cohort or cross-sectional study was used. Cohort – 11 criteria, cross-section – 8 criteria. Y = Yes, N = no, U = unclear, NA = not applicable.

Critical appraisal checklist cross-sectional studies: Item 1: Criteria for inclusion in the sample clearly designed; Item 2: Study objects and setting described in detail; Item 3: Exposure measured validly and reliably; Item 4: Objective, standard criteria used for measurement of the condition, Item 5: Confounding factors identified, Item 6: Strategies to deal with confounding factors, Item 7: Outcomes measured validly and reliably, Item 8: Appropriate statistical analysis.

Critical appraisal checklist cohort studies: Item 1: two similar groups recruited from the same population; Item 2: exposure measured similarly to assign people to both exposed and unexposed groups; Item 3: Exposure measured validly and reliably; Item 4: confounding factors identified, Item 5: strategies for confounding items stated, Item 6: Groups free of the outcome at the start of study, Item 7: Outcomes measured validly and reliably, Item 8: follow up time reported and sufficient, Item 9: Follow up complete or reasons mentioned, Item 10: Strategies to address incomplete follow-up, Item11: appropriate statistical analysis
